# Advanced Mitigation Process (AMP) for Improving Laser Damage Threshold of Fused Silica Optics

**DOI:** 10.1038/srep31111

**Published:** 2016-08-03

**Authors:** Xin Ye, Jin Huang, Hongjie Liu, Feng Geng, Laixi Sun, Xiaodong Jiang, Weidong Wu, Liang Qiao, Xiaotao Zu, Wanguo Zheng

**Affiliations:** 1 Research Center of Laser Fusion, China Academy of Engineering Physics, Mianyang, 621900, P. R. China; 2 Anhui Institute of Optics and Fine Mechanics, the university of Science and Technology of China, Hefei, Anhui 230026, P. R. China; 3 School of Physical Electronics, University of Electronic Science and Technology of China, Chengdu, 610054, P. R. China; 4 School of Materials, the University of Manchester, Manchester, M13 9PL, United Kingdom; 5 IFSA Collaborative Innovation center, Shanghai jiao tong University, Shanghai, 200240, P. R. China

## Abstract

The laser damage precursors in subsurface of fused silica (e.g. photosensitive impurities, scratches and redeposited silica compounds) were mitigated by mineral acid leaching and HF etching with multi-frequency ultrasonic agitation, respectively. The comparison of scratches morphology after static etching and high-frequency ultrasonic agitation etching was devoted in our case. And comparison of laser induce damage resistance of scratched and non-scratched fused silica surfaces after HF etching with high-frequency ultrasonic agitation were also investigated in this study. The global laser induce damage resistance was increased significantly after the laser damage precursors were mitigated in this case. The redeposition of reaction produce was avoided by involving multi-frequency ultrasonic and chemical leaching process. These methods made the increase of laser damage threshold more stable. In addition, there is no scratch related damage initiations found on the samples which were treated by Advanced Mitigation Process.

Fused silica is the isotropic glassy form of silicon dioxide. Because of its excellent optical properties, fused silica members have been widely used as the manufacturing material for optics in high power/energy laser systems such as the National Ignition Facility (NIF) in the United States^1^, the Laser MegaJoule in France^2^ and the SG series laser facility in China^3^. It is well known that surface laser induce damage ultimately limits the performance of UV wavelength region for high fluence laser applications^4^. Currently, the surface laser induce damage threshold of fused silica is still far below the intrinsic bulk limit of the material. i.e. the intrinsic laser induce damage threshold of fused silica is >100 J/cm^2 ^5. Laser induce damage is due to subsurface defects which can absorb sub-bandgap light^6^. Subsurface defects are also referred as laser damage precursors in many cases. Many kinds of laser damage precursors tend to simultaneously present in the subsurface of fused silica optics^7^. In a previous study, Suratwala *et al*. systematically isolated and identified these laser damage precursors as (1) photosensitive impurities such as cerium and cerium oxides originated from polishing process in the nanoscale Beilby layer; (2) intrinsic defects found on fracture surfaces (such as scratches) in the subsurface damage (SSD) layer; and (3) redeposited silica compounds comprising of intrinsic/extrinsic absorbing defects^8^,^9^. The laser damage occurred because of a localized optical absorption below the band gap of bulk silica when all three of these precursors were irradiated with sufficient laser fluence. Literatures show that mechanical polishing leads to two main kinds of laser precursors, i.e. impurities and shallow intrinsic defect layer. The third laser damage precursor comes from redeposition during HF etching process. This will be discussed later. Over the past years, acid etching^10^, sol-gel coatings^11^, plasma and ion beam etching^12^,^13^,^14^,^15^,^16^,^17^, as well as UV and CO_2_ laser conditioning^18^, have been tried to mitigate precursors in surface or subsurface to enhance the laser damage resistance of fused silica. Among them, HF based wet etching is an effective and conventional way^19^. Yoshiyama *et al*. showed that etching within the depth of 200 nm could improve the resistance of surface to UV laser irradiation^20^. the similar phenomenon was also observed in our previous report^10^. In addition, we have got a decrease rather than an increase in the laser induce damage threshold by further etching^10^. Recently, new report showed that the cause of decrease in damage resistance was due to redeposit of the contaminants and an effective method had been proposed to reduce the redeposition^9^. In this report, Advanced Mitigation Process (AMP) on the laser damage resistance of scratched fused silica surfaces has been investigated. The chemical leaching process was applied to make the increase of laser damage resistance stably. Additionally, more in-depth investigations are necessary to clarify the correlations between etching parameters and damage precursors to achieve more predictable and reproducible etching technology, such as comparison of scratch morphology after static etching and high-frequency ultrasonic agitation etching, comparison of laser induce damage resistance of scratched and non-scratched fused silica surfaces after HF etching with high-frequency ultrasonic agitation. And the effect of various HF-based etching processes on the laser damage resistance of fused silica substrate is also significant. And these investigations have not been reported actively.

In the previous work, we reported several new characterization techniques to analyze the correlations between damage precursors and laser induced damages, such as Time-of-Flight Secondary Ion Mass Spectrometry (TOF-SIMS). The technique can be applied to measure change of the trace elements or compounds in beibly layer because of its high sensitivity (on the order of magnitude of ppm to ppb for most species). Photo-thermal absorption of fused silica surface was measured by photo-thermal common-path interferometer^21^. In this study, mineral acid leaching and HF etching technique with multi-frequency ultrasonic agitation is used to mitigate different precursors respectively, to globally improve laser induce damage resistance of fused silica. Multi-frequency ultrasonic transducer (40, 80, 120, 140, 170, 220, 270 KHz and 0.43, 1.300 MHz) was used to reduce the redeposition. In our case, the change of the laser damage precursors in subsurface is devoted by the two kinds of characterization technologies reported in the previous work after every procedure. We also investigated comparison of scratch morphology after static etching and high-frequency ultrasonic agitation etching, comparison of laser induce damage resistance of scratched and non-scratched fused silica surfaces after HF etching with high-frequency ultrasonic agitation. And the large scale fused silica window (430 mm * 430 mm * 20) used in the high power laser facility was processed by these technologies.

## Result

**Figure 1:**
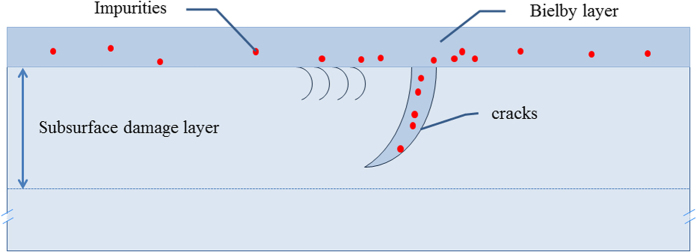
Schematic illustration of known precursors on fused silica surfaces and subsurface. The precursors are impurities in the Beilby polishing layer or cracks, the intrinsic silica defects on fracture surfaces (i.e., cracks) and redeposit of silica.

The surface and subsurface region can be described as two distinct layers:^10^ Beilby layer and subsurface damage (SSD) layer, which overlapped successively in bulk material, as schematically shown in Fig. 1. The Beilby layer consists of heavily hydrated material resulted from conventionally polish. As is well known, there are many highly absorptive photoactive impurities in the beilby layer (e.g., Ce, etc). The scratches and cracks often distribute in SSD layer under the beilby layer. As a result, the foreign matters (e.g., impurities and heavily hydrated material) often embed in the subsurface cracks. The depth of SSD layer of fused silica prepared by conventional polish technology is often more than 20 μm^8^. In a recent study, the highly absorptive photoactive impurities and the scratches was regarded as low fluence laser damage precursors. On the other hand, HF aqueous solution was used to etch SSD layer to improve laser damage resistance. The solubility of reaction product (SiF_6_^2−^) reduces because of the impurities; and that would results in redeposition on the surface of fused silica. In the case, laser damage resistance would be affected by redeposition especially for etching deeply^22^.

**Figure 2:**
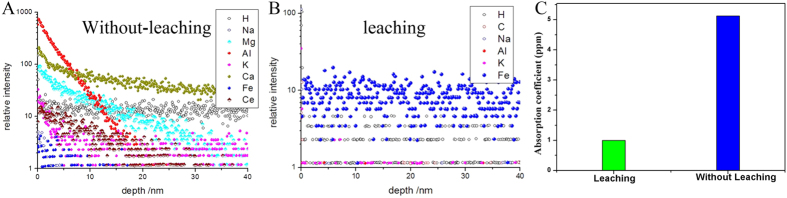
Depth profiles of impuritie elements detected in fused silica surface by TOF-SMIS: (**A**) Without leaching; (**B**) Leaching; (**C**) thermal absorption of fused silica with and without leaching.

The highly absorptive photoactive impurities can be globally processed by chemical leaching contaminants from optical surfaces. Figure 2 shows the depth profiles of impurities element detected by TOF-SIMS. Figure 2(A,B) are samples without and with chemical leaching process conditions, respectively. All of the data had been normalized with silicon particle number (counts 10,000) as a standard. Comparing Fig. 2(B) with Fig. 2 (A), it is seen that Ce element disappears absolutely and other impurities decrease at the subsurface layer of fused silica after leached in strong acid solution. The analysis of impurities defects shown in Fig. 2 suggests that metal impurities at the subsurface of fused silica nearly disappear completely after leaching in strong acid solution. On the other hand, the Fe element concentration was found to slight increase after chemical leaching. The removal mechanism of leaching in strong acid is that chemical impurities are eliminated through solubilization-diffusion. The impurity ions diffuse and solubilize because of oxidation reaction in strong acid solution. There is a balance between adsorption and desorption on the surface of substrate in the solution. Fe was one of trace elements in chemical solution. The increase of Fe element is because of the adsorption of Iron in aqueous solution of mineral acids. Even so, as reported in previous work, Fe element has a very weak influence on laser damage property in current laser damage threshold level. Cerium element is closely related to the laser damage property^23^.

In order to understand the change of thermal absorption after chemical leaching process, the thermal absorption of fused silica sample with and without leaching in strong acid solution was measured by photo-thermal common-path interferometer. As shown in Fig. 2(c), the thermal absorption of fused silica wafer was decreased from 6 ppm to 1 ppm after leaching in strong acid solution.

**Figure 3:**
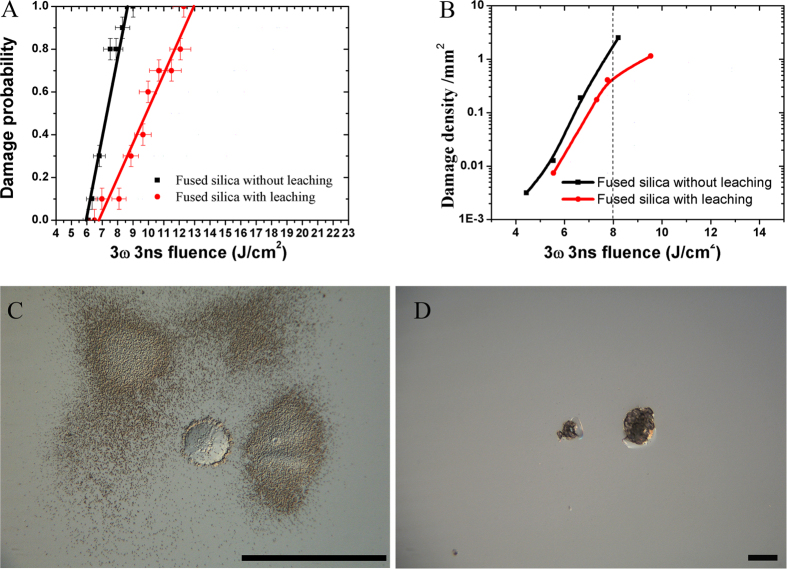
Damage probability (**A**) and Damage density (**B**) of fused silica with and without leaching. the morphology of damage site on surface of fused silica with and without leaching.

The laser induce damage threshold of fused silica also were investigated in this work. The damage density and the damage probability of fused silica wafer with and without leaching were shown in Fig. 3. The laser induced damage threshold was improved by leaching in strong acid. From the Fig. 3(A,B), it is seen that the damage density was reduced from 2.5/mm^2^ to 0.4/mm^2^ after leaching. And the 0% damage threshold increased from 5.9 J/cm^2^ to 6.8 J/cm^2^. Figure 3(C,D) show the micrographs of damage sites on fused silica surface before and after leaching. The gray haze damage sites around the big damage site in Fig. 3(A) are induced by cerium existing in the subsurface of fused silica. The damage size is about of 1 μm in diameter, as observed in high power microscopy. The gray haze damage sites are not found on surface of the fused silica which was leached by mineral acids (Fig. 3(D)). This is because leaching dissolves cerium element in the Beilby layer. Though, such strong acid leaching can only remove the surface impurities and is helpless for the subsurface cracks. Therefore, other process should be investigated to deal with the cracks. Here, HF etching technology with ultrasonic was proposed to deal with this problem.

**Figure 4:**
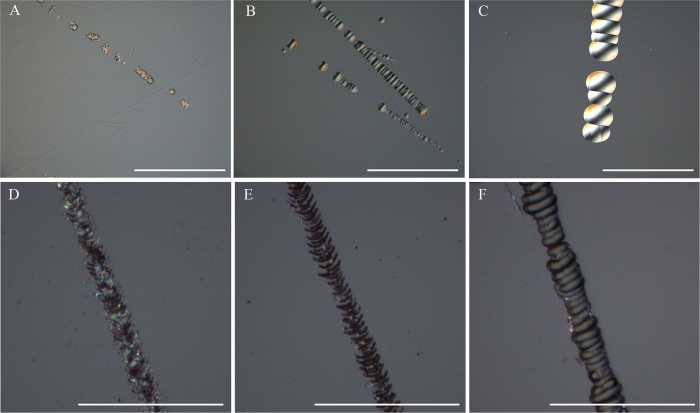
Optical micrographs of cracks generated on fused silica after HF etching with the addition of agitation (**A**, 5 min etching; **B**, 50 min etching, and **C**, 100 min etching) and static HF etching (**D**, 5 min etching; **B**, 50 min etching, and **C**, 100 min etching). The scale bar is 100 μm.

Figure 4 shows optical micrographs of scratches in the SSD layer to illustrate the change in morphology of the scratches during the HF etching process. These samples were processed by static etching and high frequency ultrasonic agitation etching, respectively. The scratches become more visible after HF etching process, as shown in Fig. 4(A–C). The scratches were also etched isotropically during the process of HF wet etching. The size of the scratches can be controlled by varying the etching duration during high frequency ultrasonic agitation etching. As presented in Fig. 4(A), the average size of the scratches is about 3 μm after 5 min HF-etching. Upon extending the etching duration to 50 min, the average of size of the scratch increases to 20 μm (Fig. 4B). After increasing etching duration further to 100 min, the scratches shown in Fig. 4(C) are about 40 μm in size. As shown in these Figures, the cracks and scratches were blunted sufficiently after 100 min etching, also leading to increased laser damage threshold. These will be discussed later.

In the next part, we focus on the difference of cracks morphology processed by static HF etching and HF etching with the addition of agitation, respectively. To the best our knowledge, this has not been demonstrated before. In the previous work, static HF etching technology is usually used to improve damage threshold. It is difficult to remove the foreign matter in the cracks for static HF etching technology. In this part, we will investigate the removing effect of foreign matter in the cracks during the static HF etching and HF etching with the addition of agitation.

The Fig. 4(D–F) show the optical micrographs of cracks from the samples etched statically. As shown in Fig. 4D, there still are many foreign matters in the cracks after 5 min HF static etching. The foreign matters in the cracks were removed obviously after 50 min and 100 min HF etching process (Fig. 4E,F). However, there are still some foreign matters which haven’t been removed in the cracks. From the comparison the Fig. 4(A–F), we can see that the foreign matters are dramatically reduced with the addition of agitation.

## Discussion

**Figure 5:**
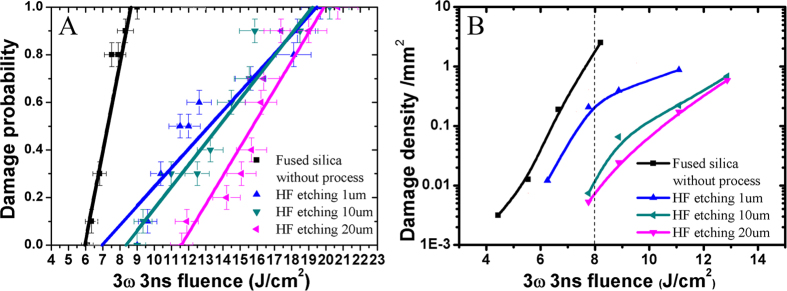
Damage probability (**A**) and Damage density (**B**) of fused silica etched different times.

In order to understand the HF etching effects on laser induce damage, we investigated laser induce damage threshold of fused silica etched different depth by HF based acid solutions. Here the damage densities tested with Raster scan are shown in Fig. 5(A), and the damage thresholds tested with 1 on 1 are shown in Fig. 5(B).

**Figure 6:**
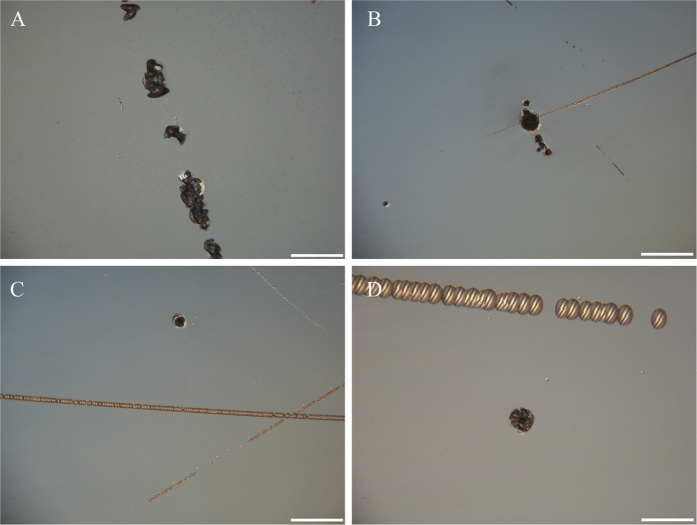
The morphology of damage site of different samples: (**A**) without process, (**B**) leaching and HF etching 5 min, (**C**) leaching and HF etching 50 min, (**D**) leaching and HF etching 100 min. The scale bar is 100 μm.

Using HF etching technology, the laser induce damage threshold can be improved with different etching time. Subsurface damage layer was removed different depths by changing the etching time. Figure 5B shows the laser damage initiation density of fused silica etched by HF-based solutions per square micrometer at the different laser fluence. After the optimized HF acid etch process, the laser damage initiation density decreased from 2.5/mm^2^ to 0.005/mm^2^ at 8 J/cm^2^ fluence. (see magenta line in Fig. 5B) The laser damage probability of these samples etched for different durations also was devoted in this work. After 5 min HF etching, the subsurface damage layer was removed 1 μm. The 0% laser induce damage threshold was 7 J/cm^2^ and 100% laser induce damage threshold was 19.3 J/cm^2^ (the bule line in Fig. 5A). The dark cyan line in Fig. 5A shows the laser damage probably of fused silica after 50 min etching. The 0% and 100% laser induce damage threshold increased to 8.4 J/cm^2^ and 19.0 J/cm^2^, respectively. In this case, the subsurface was etched 10 μm. After 100 min HF etching, the fused silica was removed 20 um, and from the magenta line in Fig. 5A, we can see the 0% laser damage threshold of the sample is 11.6 J/cm^2^; the 100% laser damage threshold increased to 19.7 J/cm^2^. The laser induce damage resistance of fused silica much depend on HF-etching process. From the Fig. 5A, it is seen that the 0% laser induce damage threshold increased with the increase of etching time. This suggests that the subsurface damage layer was removed gradually as the etching time increases. There is also other evidences that can be used to explain this issue. Figure 6 is morphology of damage site from different etching time sample. As shown in Fig. 6B, there are still scratches in the subsurface after 5 min etching. Therefore, the morphology of the damage site has similar profile with the scratches. As presented in Fig. 6C,D, after increasing etching duration further to 50 min or 100 min, all of the scratches in the subsurface damage layer appeared. The scratches cracks were blunted enough during the etching process. From them, it is seen that the damage sites always appeared on the non-scratches area. It means that the low fluence precursors e.g. scratches and cracks were mitigated enough by HF etching. To the best our knowledge, this phenomenon has not been reported in the previous work. Though, the 100% laser induce damage threshold are similar to case with different etching time. This suggests that there are high fluence precursors which cannot be mitigated by HF etching in the subsurface. These high fluence precursors would be Non-bridging Oxygen Hole Center (NBOHC) and Oxygen Deficient Center (ODC). HF etching technology was often applied to improve laser damage resistance of fused silica in previous studies. Laser damage resistance could be increased different level after different HF process^24^,^25^. Though, more than 20 um should be etched by HF acid solution for removing all the SSD layer^8^. In that case, laser damage resistance has decreased unpredictably often especially after etching deeply. That is because the reaction product deposited on surface of fused silica as discussed above. Though, multi-frequency ultrasonic and chemical leaching are used to avoid redeposition of reaction produce; and to make the increase of laser damage threshold stably in our work.

Because of isotopic HF-based wet etching, the average of the cracks increases with the increase of etching depth. As shown in Fig. 4C, the average size of cracks are several tens micrometers. And the surface roughness increases with the etching depth during HF based wet etching process. And this optics cannot be used in reality applications. Here in our case, the optimized mitigation process is acid leaching and ~20 μm SSD layer removed during HF etching.

To demonstrate the scalability of the approach, we have also processed a large optics applied in SG series facility. The fused silica optics processed using the optimized progress are 430 mm * 430 mm * 20 in size. In this progress, optics was etched 20 μm after mineral aced leaching. No damage was observed in all of the fused silica optics after 8 J/cm^2^ fluence irradiation. As shown in Fig. S1 and Table S1, the laser damage resistance of large fused silica optics was improved significantly. The amount of laser damage site in full optics at 14 J/cm^2^ decreased from 6400 to 160.

## Conclusion

In summary, we have investigated the effect of the AMP on laser induce damage resistance and damage precursor. The mineral acid leaching and HF based etching technology were used to mitigated the laser damage precursor in subsurface of fused silica, in order to improve the laser induce damage resistance. The increasing trend of laser damage threshold became more stable because of the elimination of photosensitive impurities in beilby layer after mineral acid leaching. The scratches were blunted, and the foreign matters in scratches were remarkably removed by the HF-base etching with high-frequency ultrasonic agitation. There is no scratch related damage initiations found on the samples; the laser induce damage threshold of scratches are higher than that of non-scratch area after a series of advance process. The global laser induce damage resistance were increased dramatically; The result suggest the laser damage resistance of fused silica optics can be improved significantly, thus will be useful for increasing the capacity of high power laser system.

## Materials and Methods

### Experiment section

Fused silica plates (Heraeus Suprasil 314) were ground and polished using conventional ceria polishing techniques (50 mm in diameter × 10 mm thick were produced by Z&Z Optoelectronics Tech. Co., Ltd. Chengdu). Samples were additionally leached in an acid solution (HNO_3_:H_2_O_2_ = 3:1). The fused silica substrates were then processed by HF-based etchants (HF:NH4F:H_2_O = 1:4:10) under different agitation conditions (ultrasonic, megasonic conditions at various frequencies, or statics) and etch times. And the samples were rinsed by DI water under class 100 environment conditions after leaching or HF-based etching.

Samples were first submerged in a silica tank filled with the acid solutions and agitation was generated using a multi-frequency ultrasonic transducer (Blackstone multiSONIK™ 40, 80, 120, 140, 170, 220 and 270 kHz and multiMEGTM 430, 1300 kHz). Subsequently the sample was removed and submerged in a de-ionized water rinse tank also agitated with a similar ultrasonic transducer. And then the samples were etched by HF-solutions also agitated with a similar ultrasonic transducer in a Teflon-lined tank. After HF Etched process, the samples were also rinsed by DI water in a silica rinse tank. Finally, the sample was water spray rinsed using de-ionized water and allowed to air dry. During all of the processes, samples were mounted in Teflon frames held only on the edges of the sample.

### Characteristics

Impurity element concentrations at different subsurface depth were detected using Tof-Sims with an IONTOF TOFSIMS IV apparatus. Impurity element of the sample leached by acid solutions was investigated in our case to understand the effect of acid leaching. We also measured photothermal absorption of optics surface by photo-thermal common-path interferometer based on photothermal deflection techniques. The pump beam is CW 351 nm wavelength laser. The probe beam is a He-Ne laser. They are collinear and focused through the same objective. When pump beam pass through the sample, optical absorption induces local rise of temperature. Spatial refractive index will vary due to thermal expansion. Probe beam is deflected by the modulated refractive index gradient. The deflection of the transmitted probe beam is measured by a position sensor. Photothermal techniques have a high sensitivity for small absorption. The absorption detect limit is on the order of 0.01 ppm. We measured optical thermal absorption in scanning mode with the area of 1 mm^2^ for every sample. The morphology of cracks and damage site were observed by optical microscope (olymbs)

### Laser Damage Testing

A tripled Nd:YAG laser was used at a wavelength of 351 nm in our laser irradiation test equipment. The pulse is a single longitudinal mode with about 9.3 ns (FWHM). Fluence fluctuations have a standard deviation of about ±4.5% at 351 nm. During the test, the beam is focused on the sample surface in order to achieve high fluence. The spatial beam distribution is flat Gaussian with a diameter of 3 mm for laser damage test. The modulation of irradiated area is a factor of 3.2. And the damage is always ignited at the maximum of the beam fluence. Raster scan damage test is applied to detect the laser damage density as a function of fluence using the same laser seed. The scan area is 10 cm^2^ for each fluence. In order to get the information of damage configuration, irradiated areas are detected instantaneously by a long working distance microscope with a spatial resolution of 10 μm. According to the ISO standard 11254-1, the measurement setup of laser induce damage threshold is followed the 1 on 1 method.

## Additional Information

**How to cite this article**: Ye, X. *et al*. Advanced Mitigation Process (AMP) for Improving Laser Damage Threshold of Fused Silica Optics. *Sci. Rep.* **6**, 31111; doi: 10.1038/srep31111 (2016).

## Supplementary Material

Supplementary Information
