# Supplementary material for: Advanced Mitigation Process (AMP) for Improving Laser Damage Threshold of Fused Silica Optics
Source: Sci Rep. 2016 Aug 3;6:31111. doi: 10.1038/srep31111 (PMC4971457; doi:10.1038/srep31111)
Supplement: Supplementary Information [file srep31111-s1.doc]

**Advanced** **Mitigation Process (AMP) for Improving Laser Damage Threshold of Fused Silica Optics**

**Xin Ye^1, 4^, Jin Huang1,2[[1]](#footnote-2)*, Hongjie Liu^1^, Feng Geng^1^, Laixi Sun^1^, Xiaodong Jiang^1^, Weidong Wu ^1^, Liang Qiao^4^, Xiaotao Zu^3^, Wanguo Zheng^1, 5^**

^1^ Research Center of Laser Fusion, China Academy of Engineering Physics, Mianyang, 621900, P.R. China

^2^ Anhui Institute of Optics and Fine Mechanics, the university of Science and Technology of China, Hefei, Anhui 230026, P.R. China

^3^ School of Physical Electronics, University of Electronic Science and Technology of China, Chengdu, 610054, P.R. China

^4^ School of Materials, the University of Manchester, Manchester, M13 9PL, United Kingdom

^5^ IFSA Collaborative Innovation center, Shanghai jiao tong University, Shanghai, 200240, P.R. China

Fig. S1 the photography of large size fused silica optics. Note: the size of the optics is 430mm*430mm*20mm.


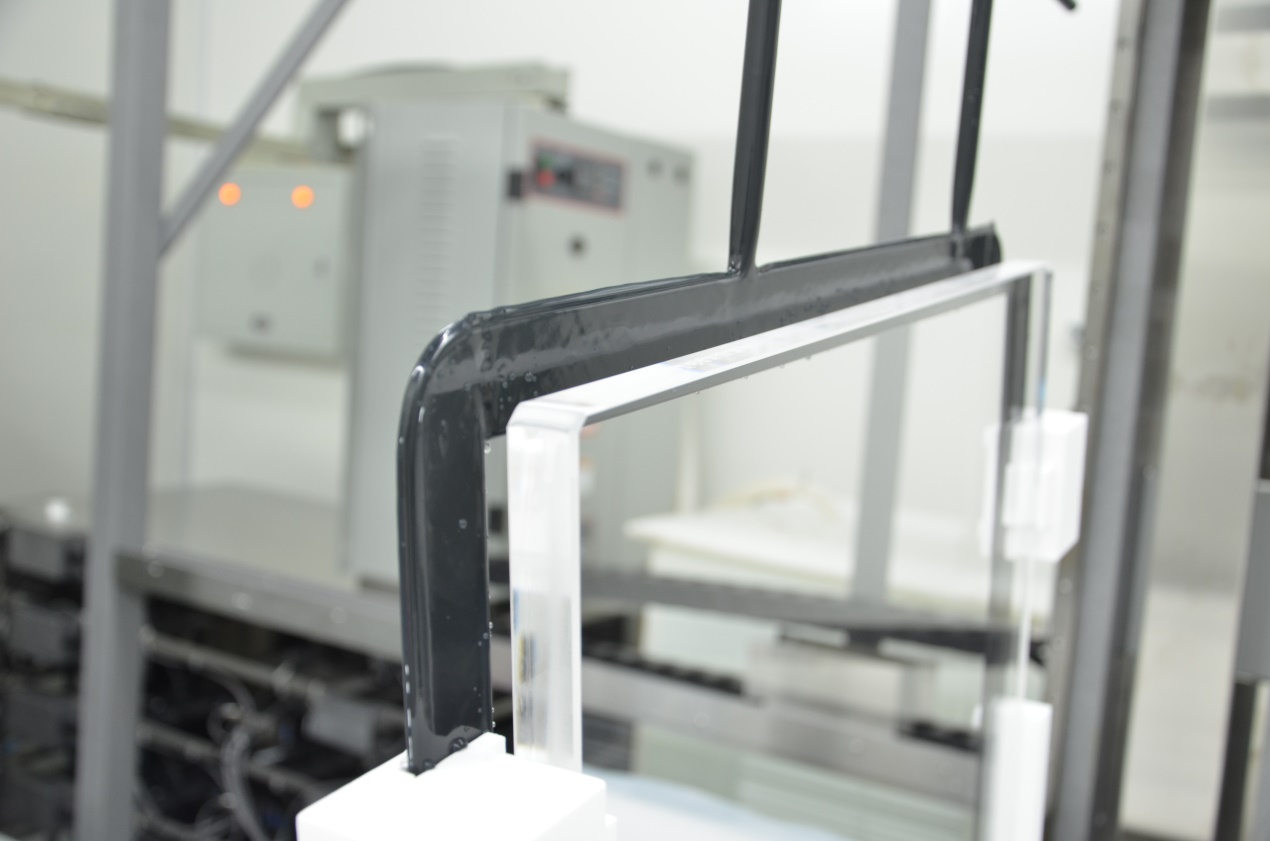


Table. S1 The amount of damage site in full size after 8J/cm2 and 14J/cm^2^ irradiation.

| Amount of damage site | 8J/cm^2^ | 14J/cm^2^ |
| --- | --- | --- |
| Without AMP | 90 | 6400 |
| AMP | 0 | 160 |

1. ^^  Correspondence should be addressed to J. Huang ([huangjin3011@163.com](mailto:huangjin3011@163.com)); X. D. Jiang ([jiangxdong@163.com](mailto:jiangxdong@163.com)) and W. G. Zheng (wgzheng_caep@sina.com)

   Tel: 86-0816-2480830; Fax: 86-0816-2480830 [↑](#footnote-ref-2)
